# Tacrolimus Drug Exposure Level and Smoking Are Modifiable Risk Factors for Early *De Novo* Malignancy After Liver Transplantation for Alcohol-Related Liver Disease

**DOI:** 10.3389/ti.2024.12055

**Published:** 2024-02-19

**Authors:** Benedict T. K. Vanlerberghe, Hannah van Malenstein, Mauricio Sainz-Barriga, Ina Jochmans, David Cassiman, Diethard Monbaliu, Schalk van der Merwe, Jacques Pirenne, Frederik Nevens, Jef Verbeek

**Affiliations:** ^1^ Department of Gastroenterology and Hepatology, University Hospitals Leuven, Leuven, Belgium; ^2^ Laboratory of Hepatology, Department of Chronic Diseases and Metabolism (CHROMETA), KU Leuven, Leuven, Belgium; ^3^ Department of Internal Medicine, Division of Gastroenterology and Hepatology, Maastricht University Medical Centre, Maastricht, Netherlands; ^4^ School of Nutrition and Translational Research in Metabolism (NUTRIM), University Maastricht, Maastricht, Netherlands; ^5^ Transplantation Research Group, Department of Microbiology, and Transplantation, Laboratory of Abdominal Transplantation, KU Leuven, University of Leuven, Leuven, Belgium; ^6^ Department of Abdominal Transplant Surgery, University Hospitals Leuven, Leuven, Belgium

**Keywords:** alcohol-related liver disease, *de novo* malignancy, calcineurin inhibitor, tacrolimus drug exposure level, liver transplantation

## Abstract

*De novo* malignancy (DNM) is the primary cause of mortality after liver transplantation (LT) for alcohol-related liver disease (ALD). However, data on risk factors for DNM development after LT are limited, specifically in patients with ALD. Therefore, we retrospectively analyzed all patients transplanted for ALD at our center before October 2016. Patients with a post-LT follow-up of <12 months, DNM within 12 months after LT, patients not on tacrolimus in the 1st year post-LT, and unknown smoking habits were excluded. Tacrolimus drug exposure level (TDEL) was calculated by area under the curve of trough levels in the 1st year post-LT. 174 patients received tacrolimus of which 19 (10.9%) patients developed a DNM between 12 and 60 months post-LT. Multivariate cox regression analysis identified TDEL [HR: 1.710 (1.211–2.414); *p* = 0.002], age [1.158 (1.076–1.246); *p* < 0.001], number of pack years pre-LT [HR: 1.021 (1.004–1.038); *p* = 0.014] and active smoking at LT [HR: 3.056 (1.072–8.715); *p* = 0.037] as independent risk factors for DNM. Tacrolimus dose minimization in the 1st year after LT and smoking cessation before LT might lower DNM risk in patients transplanted for ALD.

## Graphical Abstract

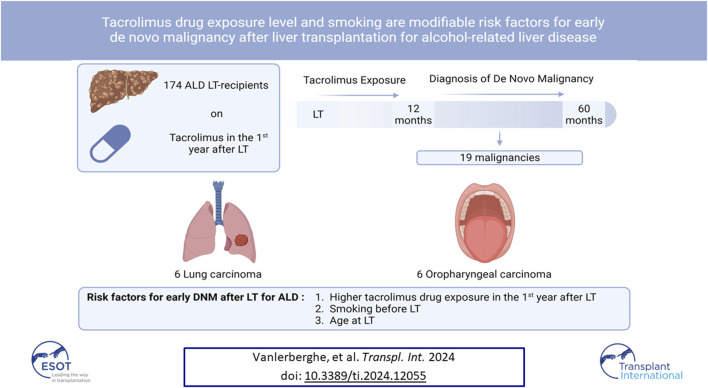

## Introduction

Alcohol-related liver disease (ALD) is the primary indication for liver transplantation (LT) in Europe and the United States [1]. *De novo* malignancies (DNM) are the leading cause of mortality in patients transplanted for ALD [2–4], a population with an increased risk of DNM compared to patients who received a LT for other indications [5]. This might be attributed to the oncogenic effects of long-term alcohol consumption [6, 7] and the high prevalence of tobacco use in patients with ALD [8]. Another risk factor for DNM is the immunosuppressive therapy patients receive after LT [9, 10]. Calcineurin inhibitors (CNIs) are the most frequently used agents for long-term immunosuppression and tacrolimus is currently the most frequently used CNI [1]. Tacrolimus reduces the risk of allograft rejection after LT by inhibiting calcineurin, which leads to decreased cytokine transcription, particularly interleukin-2 (IL-2), and reduces T-cell proliferation [11]. Tacrolimus and its immunosuppressive properties are also associated with the development of DNM due to diminished immunosurveillance [12]. However, studies analyzing the risk of tacrolimus exposure and trough levels on DNM development after LTx are scarce [13–15]. These few studies did not specifically analyze ALD LT recipients [13, 14] and did not examine the effect of lifetime tobacco consumption and alcohol relapse after LT, factors that might have a strong contributive effect on malignancy formation. Furthermore, these studies assessed the lifetime DNM risk after LT based on 1st year of tacrolimus drug exposure. However, the 1st year exposure might only reflect the DNM risk within a shorter time after LT. Therefore, in this study we assessed the risk factors for DNM in the 1st 5-year period after LT for ALD, with specific emphasis on tacrolimus drug level in the 1st year post-LT and other modifiable risk factors such as smoking and alcohol use.

## Patients and Methods

### Patient Characteristics

We analyzed the Liver Transplantation Cohort from the University Hospitals KU Leuven for adult patients (age ≥ 18 years) transplanted for ALD between 1990 and October 2016. Diagnosis of ALD was made based on the patient’s history of excessive and habitual alcohol consumption, clinical and laboratory findings, histology of the explanted liver, and exclusion of other causes of liver disease (viral hepatitis, autoimmune hepatitis, hereditary hemochromatosis, Wilson’s disease, primary biliary cholangitis, and primary sclerosing cholangitis), and based on the consensus of the multidisciplinary medical team. Patients with less than 12 months follow-up post-LT, a DNM in the 1st year post-LT or patients who did not receive tacrolimus during the 1st 12 months after LT were excluded from the analysis. Patients with unknown smoking habits (no information available on smoking history, i.e., pack years smoked before LT) were also excluded from the analysis. We retrospectively collected data on general patient characteristics, type and timing of DNM, excluding non-melanoma skin cancer and recurrence of hepatocellular carcinoma as events. DNM were reported as events if they occurred between 12 and 60 months of follow-up. Positive smoking history (defined as ≥ 1 pack year pre-LT), absolute number of pack years (PY) smoked before LT and active smoking at LT were extracted from patient’s medical files. Active smoking was defined as smokers who still had an active smoking habit when admitted to the hospital for the LT procedure. Alcohol relapse was defined as relapse with any alcohol use.

In the work-up before LT, each patient is systematically screened for malignancies. Patients receive a gastroscopy, colonoscopy, chest x-ray, liver MRI and/or CT abdomen, abdominal ultrasound, and an assessment by an ear-nose-throat specialist and dermatologist. Female LT candidates also receive a mammography and a gynecological ultrasound. After LT for ALD, patients do not receive additional systematic screening for DNM aside from the Belgian government’s population screening for colon and breast cancer.

### Immunosuppressive Regimen

After LT, the standard immunosuppressive protocol consists of tacrolimus, an antimetabolite, and corticosteroids. After 3 months, corticosteroids are discontinued. If possible, antimetabolite is discontinued after 12 months. The used antimetabolites are mycophenolate, and azathioprine in the minority of patients. Deviations from the protocol are only performed on clinical indication. Tacrolimus trough levels are analyzed daily during hospital admission directly following LT. After hospital discharge, trough levels are determined twice a week during the first month, every 2 weeks during the first 3 months, three monthly from 3 months to 1 year post-LT and per 3–6 months thereafter. On clinical indication, tacrolimus trough levels are analyzed more frequently.

### Statistical Analysis

Normality was checked by the Shapiro-Wilk test. Qualitative variables were compared using the χ^2^ test. Normally distributed values are presented as means with 95% confidence intervals (95% CI) or standard deviation (SD) and were compared using an independent t-test. Non-normally distributed values are presented as medians with interquartile range (IQR) and were compared using the Mann-Whitney U and Kruskal-Wallis tests.

Hazard ratios (HR) for the risk of DNM between twelve and 60 months after LT were evaluated by Cox proportional hazards regression. Patients were considered at risk from twelve to 60 months post-LT or until reaching the study endpoints (diagnosis of DNM or death). Patients lost to follow-up were censored at time of loss from data analysis. A proportional hazard model was performed with categorical, continuous, and time-dependent covariates to identify risk factors. Additional covariates were selected by expert opinion and based on literature [13, 14]. The proportional hazard assumption was tested for each covariate by correlation testing of Schoenfeld residuals and rank time; only covariates without significant correlation were included (see Supplementary Table S1). Multivariate analysis was performed by backward elimination with a selection criterion of 0.100. Statistical analysis was performed by SPSS v.28 (SPSS Inc., Chicago, IL, United States). Statistical significance was defined as *p* ≤ 0.05.

Total tacrolimus drug exposure level (TDEL) was based on the area under the curve of tacrolimus trough levels in µg/L [analyzed in R by PKCNA package (linear)] and corrected by the trapezoidal rule as previously described by Vivarelli et al. [16].

## Results

### Patient Characteristics

Within the study period, 317 patients were transplanted for ALD at our center, of which 174 were included in the analysis. Patients who had a follow-up of 12 months or less (*n* = 23; 22 died within the 1st year post-LT and one patient was lost to follow-up at 11 months post-LT), patients who were diagnosed with a DNM within 12 months after LT (*n* = 11) and patients who did not receive tacrolimus in the 1st year after LT (*n* = 46) were excluded. 56 patients were excluded because there was insufficient information available on their smoking habits. Seven patients were excluded because of missing data on tacrolimus trough levels in the 1st year post-LT. All included patients were transplanted after December 1998.

During the 1st year after LT, 117 (67.2%) patients received *Prograft* and 57 (32.8%) *Advagraf*. 144 (82.8%) patients received mycophenolate and 20 (11.5%) patients received azathioprine as an antimetabolite. The median age at LT was 59.5 years (IQR: 54.0–65.0) (Table 1). Median follow-up was 91 months (IQR: 65.0–143.0).

**TABLE 1:** General characteristics of patients at liver transplantation diagnosed with a *de novo* malignancy compared to those not diagnosed with a *de novo* malignancy.

	Overall (*n* = 174)	DNM (*n* = 19)	No DNM (*n* = 155)	*p*-value
Male sex (%)	133 (76.4)	17 (89.5)	116 (74.8)	0.156
Median age in years (IQR)	59.5 (54.0–65.0)	66.0 (58.0–68.0)	59.0 (54.0–64.0)	0.145
HCC on explant (%)	70 (40.2)	10 (52.6)	60 (38.7)	0.243
Combined transplant (%)	14 (8.0)	2 (10.5)	12 (7.7)	0.674
Median Child-Pugh Score at LT (IQR)	10.0 (8.0–11.0)	9.0 (7.0–11.0)	10.0 (8.0–11.0)	0.976
Median MELD-Na at LT (IQR)	18.0 (11.0–24.0)	18.0 (9.0–22.0)	18.0 (11.8–24.0)	0.805
Smoking history (%)	112 (64.4)	18 (94.7)	94 (60.6)	0.003*
Active smoking (%)	44 (25.3)	8 (42.1)	36 (23.2)	0.074
Median pack years of patients with a smoking history (IQR)	30.0 (20.0–43.8)	42.5 (35.0–50.3)	30.0 (20.0–40.0)	0.004*
MFM (%)	144 (82.8)	14 (73.7)	130 (83.9)	0.267
Azathioprine (%)	20 (11.5)	4 (21.1)	16 (10.3)	0.166
No antimetabolite (%)	10 (5.7)	1 (5.3)	9 (5.8)	0.923
mTOR inhibitor ^ǂ^ (%)	10 (5.7)	0 (0)	10 (6.5)	0.254
Basiliximab at LTx (%)	15 (8.6)	1 (5.3)	14 (9.0)	0.581
Alcohol relapse after LT (%)	60 (34.5)	6 (31.6)	54 (34.8)	0.778
Median Donor age in years (IQR)	57.0 (44.0–64.0)	57.0 (38.0–66.0)	57.0 (45.0–64.0)	0.810
Cold ischemia time (hours: minutes)	7:36 (5:29–9:08)	7:17 (5:00–9:07)	7:45 (5:30–9:11)	0.470

LT, liver transplantation; DNM, *de novo* malignancy; IQR, interquartile range; HCC, hepatocellular carcinoma; MFM, mycophenolate mofetil; mTOR, mammalian target of rapamycin inhibitor; ǂ: number of patients who received an mTOR inhibitor combined witch CNI for at least 1 month in the 1st year after LTx, * = statically significant.

Bold values denote statistical significance.

Of the total group, 112 (64.4%) patients had a positive smoking history, of which 44 (25.3% of the total group) actively smoked at the time of transplantation (Table 1). 60 patients (34.5%) had a relapse of any alcohol use after LT. 47 patients (78.3% of the relapsers) relapsed within 5 years after LT, with a median time until relapse of 15.0 months (IQR: 4.0–32.0).

### *De Novo* Malignancy

19 (10.9%) patients were diagnosed with a DNM between 12 and 60 months after LT. Characteristics of patients diagnosed with a DNM compared to those who did not can be found in Table 1. Median time until diagnosis of DNM was 46 months (IQR: 18.0–56.0). 17 patients developed a solid organ malignancy with oropharyngeal (*n* = 6) and lung carcinoma (*n* = 6) being the most prevalent (Table 2). One patient developed a simultaneous lung and oropharyngeal carcinoma at 54 months post-LT. Of the 17 solid organ DNMs, 5 (29.4%) patients had metastatic disease, 9 (52.9%) had only local disease and in 3 (17.6%) patients staging at the time of diagnosis was not reported. Four of the six patients diagnosed with lung carcinoma died from the malignancy during follow-up. The other two only had local disease at diagnosis and were alive after 1 and 23 months of follow-up. Two out of six patients diagnosed with an oropharyngeal carcinoma died because of the carcinoma, yet all diagnoses were made in a non-metastatic stage. All patients diagnosed with a lung or oropharyngeal malignancy had a smoking history. Two patients have developed hematological malignancies (1.1%) being a post-transplant lymphoproliferative disease and a plasmacytoma. One patient developed a soft tissue tumor (Kaposi sarcoma).

**TABLE 2:** Types of *de novo* malignancies diagnosed after liver transplantation.

Type of DNM	Time diagnosis (months after LTx)	Stage at diagnosis	CNI	Pack years before LTx	Follow-up after DNM (months)	Cause of death
Lung carcinoma (spinocellular)	16	T4N3M1	Tacrolimus	60	20	Lung carcinoma
Lung carcinoma (spinocellular)	21	T3N0M1	Tacrolimus	51	90	Lung carcinoma
Lung carcinoma (adenocarcinoma)	25	TN*n/a*M1	Tacrolimus	35	13	Lung carcinoma
Lung carcinoma (undifferentiated)	45	*n/a*	Tacrolimus	45	1	Lung carcinoma
Lung carcinoma* (spinocellular)	54	T2N0M0	Tacrolimus	60	23	
Lung carcinoma (spinocellular)	57	T1N0M0	Tacrolimus	50	1	
Oropharyngeal carcinoma	13	T1N1M0	Tacrolimus	50	11	Oropharyngeal carcinoma
Oropharyngeal carcinoma	17	T1N0M0	Tacrolimus	40	52	Oropharyngeal carcinoma
Oropharyngeal carcinoma	18	T2N0M0	Tacrolimus	35	125	
Oropharyngeal carcinoma	25	T1N0M0	Tacrolimus	30	63	Pneumonia
Oropharyngeal carcinoma*	54	T1N1M0	Tacrolimus	60	23	
Oropharyngeal carcinoma	58	*n/a*	Tacrolimus	40	31	
Colorectal carcinoma	47	TN*n/a*M1	Tacrolimus	30	6	Colorectal carcinoma
PTLD	56	IVa	Tacrolimus	12	8	PTLD
TCC (Bladder)	46	TxG1	Tacrolimus	45	59	COPD
TCC (Bladder)	57	TN*n/a*M1	Tacrolimus	36	1	TCC (Bladder)
Esophagus carcinoma	49	*n/a*	Tacrolimus	60	28	
Ovarian carcinoma	60	FIGO II	Tacrolimus	50	27	
Plasmacytoma	13		Tacrolimus	0	218	Alcoholic cirrhosis
Sarcoma Kaposi	48	High Grade	Tacrolimus	40	5	Sarcoma

DNM, *de novo* malignancy; LTx, liver transplantation; CNI, calcineurin inhibitor; n/a, not available; PTLD, post-transplant lymphoproliferative disorder; COPD, chronic obstructive pulmonary disease; TCC, transitional cell carcinoma; * DNM diagnosed in the same patient.

**FIGURE 1:**
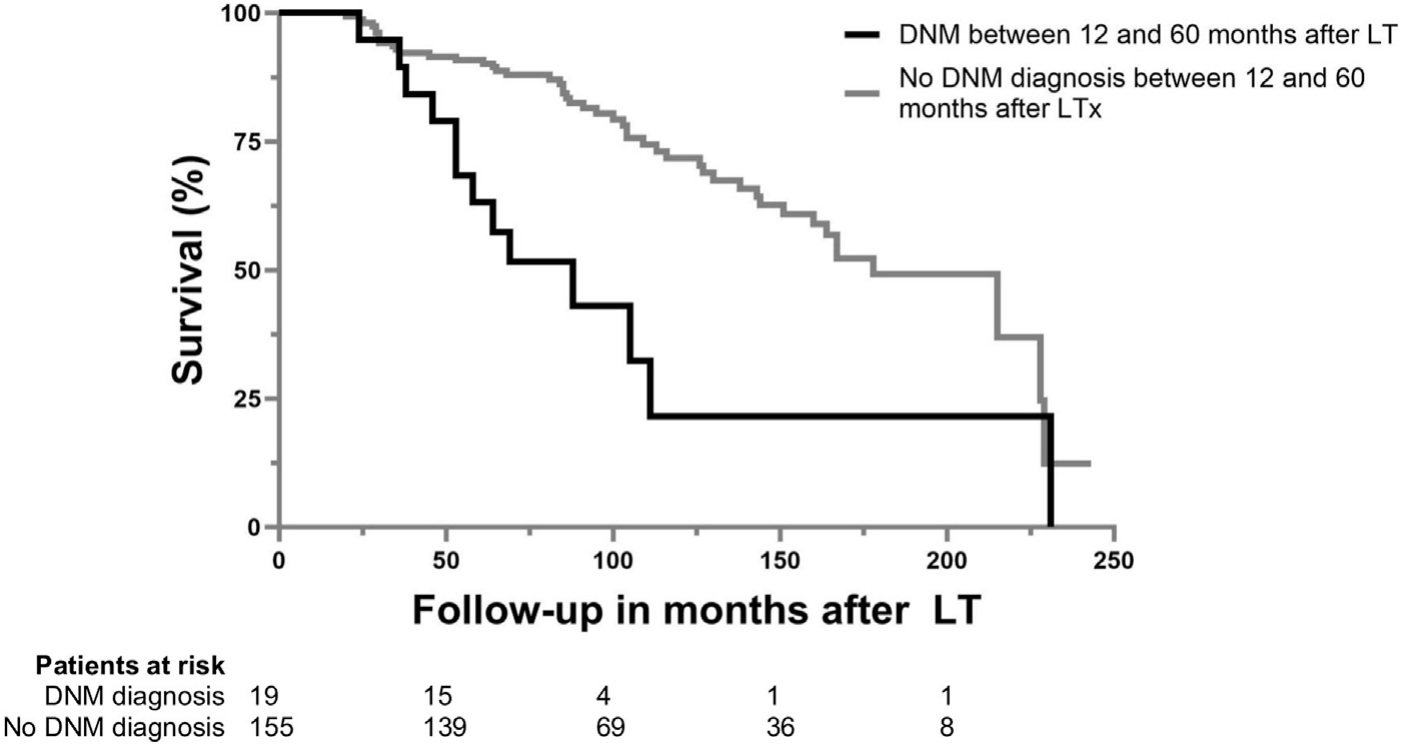
Patient survival after LTx for ALD based on the diagnosis of *de novo* malignancy between 12 and 60 months after LT.

Patients diagnosed with a DNM between 12 and 60 months after LT had a higher mortality than those not diagnosed with a DNM [HR: 2.981 (95% CI: 1.573–5.652); *p* < 0.001)] (see Figure 1). Of patients diagnosed with a DNM, 13 (68.4%) died during follow-up, of which 10 died due to DNM. The overall 60 months survival was 81.0% (*n* = 141), and was significantly higher in the group without DNM (83.9%) compared to the group with a DNM (57.9%) (*p* = 0.006), corresponding with a shorter median follow-up in the DNM group than in the non-DNM group [69.0 months (IQR: 53.0–89.0) vs. 95.0 (IQR: 70.0–145.0); *p* = 0.020].

### Risk Factors for Developing DNM

#### Tacrolimus Drug Exposure Level

The mean tacrolimus drug exposure level in the 1st year post-LT was 7.41 μg/L (SD. 1.60) and was higher in patients who developed a DNM within 5 years after LT (8.14 μg/L; SD. 1.56) compared to patients who did not (7.31 μg/L; SD. 1.59) (*p* = 0.016). Univariate cox regression analysis identified TDEL as a risk factor for DNM between 12 and 60 months after LTx [HR: 1.357 (95% CI: 1.027–1.790); *p* = 0.031].

When analyzing the ROC of TDEL plotted against DNM prevalence, a TDEL of 6.94 μg/L had the highest discriminative value. 15 out of 98 patients (15.3%) with a TDEL above 6.94 μg/L developed a DNM compared to 4 out 76 patients (5.3%) with a TDEL lower than 6.94 μg/L. Also when implementing TDEL as a categorical value using the cut-off of 6.94 μg/L, TDEL remained a significant risk factor for DNM [HR: 3.009 (95% CI: 1.000–9.067); *p* = 0.050].

#### Other Risk Factors for DNM

18 (94.7%) patients with a DNM had a positive smoking history (≥1 pack year pre-LT) compared to 94 (60.6%) of patients who did not develop a DNM (*p* = 0.003). Patients who smoked and were diagnosed with a DNM had a higher tobacco consumption at LT than those without a DNM diagnosis [median pack years: 42.5 (IQR: 35.0–50.3) vs. 30.0 (IQR: 20.0–40.0); *p* = 0.004].

Univariate cox regression analysis identified age at LT [HR: 1.115 (95% CI: 1.039–1.197), *p* = 0.002], smoking history [HR: 11.136 (95% CI: 1.486–83.451); *p* = 0.019] and number of pack years pre-LT [HR: 1.024 (95% CI: 1.010–1.038); *p* = 0.001] as risk factors for developing DNM in the 1st 5 years after LT. In multivariate analysis, only pack years and not smoking history (defined as ≥ 1 pack year pre-LT) was included to avoid multicollinearity (Table 3).

**TABLE 3:** Univariate and multivariate cox proportional hazard regression for risk on *de novo* malignancy between 12 and 60 months after liver transplantation (174 patients) in patients under tacrolimus in first-year after liver transplantation.

	Univariate	Sig.	Multivariate	Sig.
	HR (95% CI)	*p*-value	HR (95% CI)	*p*-value
Age at LT (mean-centered)	1.115 (1.039–1.197)	0.002*	1.158 (1.076–1.246)	<0.001*
Male sex	2.971 (0.686–12.871)	0.145		
Alcohol relapse (T)	0.681 (0.197–2.356)	0.544		
Smoking history	11.136 (1.486–83.451)	0.019* ^**ǂ**^		
Pack years pre-LT	1.024 (1.010–1.038)	0.001*	1.021 (1.004–1.038)	0.014*
Active smoking at LT	2.356 (0.947–5.860)	0.065	3.056 (1.072–8.715)	0.037*
TDEL	1.357 (1.028–1.790)	0.031*	1.710 (1.211–2.414)	0.002*
MFM vs. Azathioprine	0.494 (0.163–1.502)	0.214		

HR, hazard ratio; CI, confidence interval; LT, liver transplantation; TDEL, tacrolimus drug exposure level; MFM, mycophenolate mofetil; * = statically significant, ǂ = not included in multivariate analysis, T = time-dependent variable.

Bold values denote statistical significance.

Multivariate analysis identified age at LT [HR: 1.158 (95% CI: 1.076–1.246); *p* < 0.001], number of pack years pre-LT [HR: 1.021 (95% CI: 1.004–1.038); *p* = 0.014], active smoking at LT [HR 3.056 (95% CI: 1.072–8.715); *p* = 0.037] and TDEL in the 1st year after LT [HR: 1.710 (95% CI: 1.211–2.414); *p* = 0.002] as independent risk factors for developing DNM in the 1st 5 years after LT (Table 3). The use of mycophenolate mofetil compared to azathioprine, sex, and any alcohol relapse within 5 years after LT were not associated with a higher risk on DNM after LT.

#### Rate of Liver Graft Rejection

21 (12.1%) patients of the total group developed an acute cellular rejection (ACR) within the first 30 days after LT. Patients with a graft rejection in the first month had a higher TDEL in the first year after LT [mean TDEL: 8.10 μg/L (SD. 1.69) vs. 7.31 μg/L (SD. 1.57); *p* = 0.034]. This resulted in a trend towards a higher number of DNM in the ACR-group [*n* = 4 (19.0%)] vs. the non-ACR-group [*n* = 15 (9.8%)], but this did not reach statistical significance (*p* = 0.203).

## Discussion

Identifying modifiable risk factors for DNM in patients transplanted for ALD is crucial to optimize their outcome. In our study, we found that in patients transplanted for ALD, tacrolimus drug exposure level (TDEL) in the 1st year after LT, number of pack years before LT, active smoking at LT and older age at LT are independent risk factors for the development of early DNM within 12 and 60 months after LT. Hereby, we provide the first evidence on the impact of TDEL in the 1st year after LT for ALD on the occurrence of early DNM, taking into account a detailed analysis on smoking habits.

The development of malignancies after LT is a complex interaction between an individual’s risk on DNM based on genetic predisposition, exposure to carcinogenic viruses, previous and current behavior such as smoking and alcohol use, and immunosuppressive therapy [12]. The observed association of TDEL and DNM is in line with the findings of Carenco et al. [14] and Rodriguez-Perálvarez et al. [13], although these two studies were conducted in LT patients transplanted not merely for ALD, and used DNM at any time point after LT as outcome parameter. Taken together, these observations underline the potential of optimizing tacrolimus drug exposure in clinical care. Prospective studies should focus on identifying protocols aiming for the minimally acceptable tacrolimus through level in order to lower the DNM risk, without increasing allograft rejection. This might be achieved by the use of induction therapy and/or the concurrent use of other immunosuppressive agents such as mammalian target of rapamycin (mTOR) inhibitors. However, there is conflicting evidence on the impact of mTOR inhibitors on DNM and if their underlying effect is associated with their antiproliferative properties or their tacrolimus-saving effect [17]. The development of pharmacometric tools to accurately predict TDEL based on daily tacrolimus dosage, patient characteristics and concomitant medication could be another approach, which we are currently studying in our center [18, 19]. In our study, we observed a higher TDEL in the 1st year after LT in patients that experienced an acute cellular rejection, but could not associate low TDEL with an increased risk for rejection.

Identifying tacrolimus trough level cut-offs that are associated with an increased risk for DNM, yet not with an increased risk on rejection, would be helpful in the follow-up and care of our LT patients. Within our cohort the cut-off over which DNM risk was disproportionately increased was 6.94 μg/L. However given the retrospective nature of our study this cut-off should not be extrapolated to other cohorts. Prospective studies are needed to further establish cut-offs that are usable in clinical care.

Patients with a positive smoking history (≥1 pack year pre-LT) had a substantially higher risk of developing DNM. Accordingly, all patients with lung or oropharyngeal carcinoma, the most frequent DNMs, had a positive smoking history. There seemed to be a dose-related effect since for every pack year smoked before LT there was an increase in DNM risk (HR: 1.021). Furthermore, patients who actively smoked at LT had an increased risk of DNM independent from the number of pack years they smoked before LT or TDEL. Together, these data stress the importance of smoking cessation in the pretransplant period and support the implementation of smoking cessation programs [20]. Since alcohol is a carcinogen [21], we expected that alcohol relapse could lead to a higher incidence of DNM. However, we did not find a higher risk for DNM after any alcohol relapse, in line with other studies [22]. This could be explained by the higher risk of liver-related disease and mortality after alcohol relapse, which could occur before the development of DNM [1]. Age at LT was an independent risk factor for developing DNM. In our cohort and other centers [1], over the last decades, patients have become older at the time of LT due to a switch in focus from chronological to biological age as an eligibility criterium for LT listing [1]. Transplanting older patients will only increase DNM incidence post-LT in the future, underscoring the relevance of our findings and the need for measures to lower DNM risk.

In our study, lung carcinomas were frequently diagnosed in a metastatic setting, whereas oropharyngeal were not. Screening could be implemented in clinical practice to diagnose lung carcinoma in curative stages, which was the focus of several studies. Renaud et al. compared an intensive screening program (yearly chest CT and clinical examination by an otorhinolaryngologist versus chest CT every 5 years) in patients transplanted for ALD who continued to smoke after LT [23]. They found that 63.6% of patients underwent a curative treatment of lung carcinoma in the intensive screening program versus only 26.3% in the standard screening program (*p* = 0.062) [23]. There was no difference in curative treatment of oropharyngeal tumors between the groups [100% vs. 87.5% (*p* = 0.498)] [23]. These findings are comparable with our results, where in a clinical setting without systematic post-LT screening, all diagnoses of oropharyngeal tumors were made with a possibility of curative treatment. Other studies analyzing the impact of malignancy screening after LT also showed that screening led to diagnosing DNM in earlier stages with higher rates of curative treatment [24, 25]. However, these studies were not limited to ALD LT recipients, therefore caution is warranted to extrapolate these findings. We propose that future studies assessing the benefit of DNM screening post-LT should primarily focus on ALD patients who have a proportionally higher risk for DNM. Based on our data and those of Renaud et al. a yearly screening with chest CT for lung carcinoma might be beneficial in active smokers [23], and potentially in all patients with a smoking history.

Although we provided a detailed assessment of the effect of TDEL and smoking habits on the risk of DNM in patients transplanted for ALD, the retrospective nature of our study is a limitation. On the other hand, the single-center approach enables assessment of DNM risk in ALD patients that underwent similar work-up, treatment and follow-up regarding LT, yet this also implies that our data need external validation.

In conclusion, our study identified high tacrolimus drug exposure levels in the first year post-LT and smoking as significant risk factors for early DNM after LT for ALD. Therefore, tacrolimus overexposure should be avoided and more efforts for smoking cessation should be initiated in these patients. Future studies are needed to assess the value and cost-benefit of systematical DNM screening after LT.

## Data Availability

The raw data supporting the conclusion of this article will be made available by the authors, without undue reservation.
