# Supplementary material for: Tacrolimus Drug Exposure Level and Smoking Are Modifiable Risk Factors for Early De Novo Malignancy After Liver Transplantation for Alcohol-Related Liver Disease
Source: Transpl Int. 2024 Feb 19;37:12055. doi: 10.3389/ti.2024.12055 (PMC10909820; doi:10.3389/ti.2024.12055)
Supplement: Supplementary file 1 [file Table1.docx]

***Supplementary Table 1: Goodness of fit model: assessing proportional hazard assumption by analyzing zero-correlation coefficient of partial residuals of covariates and rank value of survival time.***

|  | *P- value* |
| --- | --- |
| Age at LTx (mean-centered) | .082 |
| Male sex | .950 |
| Smoking history | .111 |
| Pack years pre-LTx | .647 |
| Active smoking at LTx | .887 |
| TDEL | .348 |
| MFM vs. Azathioprine | .647 |

**Legend:** LT: liver transplantation, TDEL: tacrolimus drug exposure level, MFM: mycophenolate mofetil
